# Helical axis analysis to quantify humeral kinematics during shoulder rotation

**DOI:** 10.1080/23335432.2019.1597642

**Published:** 2019-04-01

**Authors:** Corrado Cescon, Marco Barbero, Marco Conti, Francesco Bozzetti, Jeremy Lewis

**Affiliations:** a Rehabilitation Research Laboratory 2rLab, Department of Business Economics, Health and Social Care, University of Applied Sciences and Arts of Southern Switzerland, Manno, Switzerland; b MedSport Sports Medicine, Como, Italy; c Department of Allied Health Professions, School of Health and Social Work, University of Hertfordshire, College Lane, Hatfield, UK

**Keywords:** Helical axis, shoulder, joint, kinematics

## Abstract

Information pertaining to the helical axis during humeral kinematics during shoulder rotation may be of benefit to better understand conditions such as shoulder instability. The aim of this study is to quantify the behavior of humeral rotations using helical axis (HA) parameters in three different conditions. A total of 19 people without shoulder symptoms participated in the experiment. Shoulder kinematics was measured with an optoelectric motion capture system. The subjects performed three different full range rotations of the shoulder. The shoulder movements were analyzed with the HA technique. Four parameters were extracted from the HA of the shoulder during three different full-range rotations: range of movement (RoM), mean angle (MA), axis dispersion (MDD), and distance of their center from the shoulder (D). No significant differences were observed in the RoM for each condition between left and right side. The MA of the axis was significantly lower on the right side compared to the left in each of the three conditions. The MDD was also lower for the right side compared to the left side in each of the three conditions.The four parameters proposed for the analysis of shoulder kinematics showed to be promising indicators of shoulder instability.

## Introduction

The glenohumeral joint caSSn produce extremely fast and precise movement across large ranges to permit the upper limb to perform complex functional tasks (Roach et al. 2013). Both passive and active structures of the shoulder contribute to maintain the stability between the humeral head and the glenoid fossa (Degen et al. 2013; Murray et al. 2013). During active movements of the shoulder, the rotator cuff and shoulder ligaments stabilize the glenohumeral joint by limiting humeral head translations in supero-inferior direction and in antero-posterior direction (Sahara et al. 2007). In addition, the morphology of the shoulder complex allows by a wide spectrum of movements, where ligaments and muscles guarantee its stability (Halder et al. 2000; Blaimont et al. 2006). For this reason, neuromuscular control plays an important role, minimizing the displacement of the center of rotation of the shoulder during upper limbs movements (Doorenbosch et al. 2003; Blaimont et al. 2006).

The application of the helical axis (HA) has been suggested as a method of investigating joint kinematics (Cattrysse et al. 2007; Cripton et al. 2001; Helena Grip et al. 2008; Woltring et al. 1985). Using the HA, the motion of a body segment is defined as a rotation about and translation along a single axis. The position and the orientation of the HA may be used to describe the quality of a movement using 3D kinematics approach.

HAs dispersion may be used as an index of joint stability and research has been conducted in the ankle, knee and cervical regions (Barbero et al. 2017; Cescon et al. 2014; Graf and Stefanyshyn 2012; Grip and Häger 2013).

In a recent publication, the convex hull area (a parameter describing the dispersion of a number of points laying on a plane) has been computed to quantify the change in position of a group of HA, and the mean angle has been computed to quantify their change in orientation (Cescon et al. 2014).

It would be of value to add to the current body of knowledge pertaining to the HA in other regions by evaluating the shoulder FHAs behavior in different conditions of neuromuscular control, such as with or without visual feedback, as in case of dominant versus non-dominant upper limb (Heuer 2007; Sachlikidis and Salter 2007; Assi et al. 2013). In addition, the analysis of a controlled condition such as a constrain that would limit the elbow displacement during humeral rotation, would highlight the behavior of the glenohumeral complex. The acquisition of such knowledge may be beneficial in future research investigating clinical conditions involving the shoulder such as instability which has been associated with displacement of the humeral head on the glenoid fossa (REF). To our knowledge, no study has been conducted to quantify the kinematics of the shoulder using the HA approach during a dynamic task, one that requires a contribution of both the active and passive stabilizers of the shoulder.

As such, the aim of this pilot study was to analyze the HA in three different tasks, and comparing dominant and non-dominant side, in people without symptoms.

## Materials and methods

This pilot study was conducted at Rehabilitation Research Laboratory 2rLab of University of Applied Sciences and Arts of Southern Switzerland. The data collection was performed between October 2015 and February 2016.

### Subjects

Nineteen healthy volunteers (7 males, 12 females, age: 23.2 ± 2.7 years) participated in the experiment signing an informed consent form in accordance to the Declaration of Helsinki. Exclusion criteria was the presence of shoulder pain in the last six months.

### Equipment

Shoulder kinematics was measured with an optoelectric motion capture system (Optitrack, OR, USA) including six cameras. The frame rate of the optoelectric system was 120 fps. The infrared cameras were fixed to a square metal frame with four meters edge length. The metal frame was attached to the ceiling at 2.4 m height. The cameras were positioned so that they formed an hexagon and were all pointing to the center of the square, one meter above the ground level. An additional optical camera was positioned 3 m behind the subject on a stative at 1.2 m height and used to provide a visual feedback of arm position to the subjects on a 19” PC screen positioned on a table 2 m in front of the subjects.

The chosen camera setup was evaluated using three markers placed on a standard wand (50 cm). The marker position accuracy was estimated to be below 1 mm by the Motive software (Optitrack, OR, USA) used to acquire the data. The markers were plastic spheres of 10 mm diameter, covered with reflecting material and attached to a rubber support.

**Figure 1.**
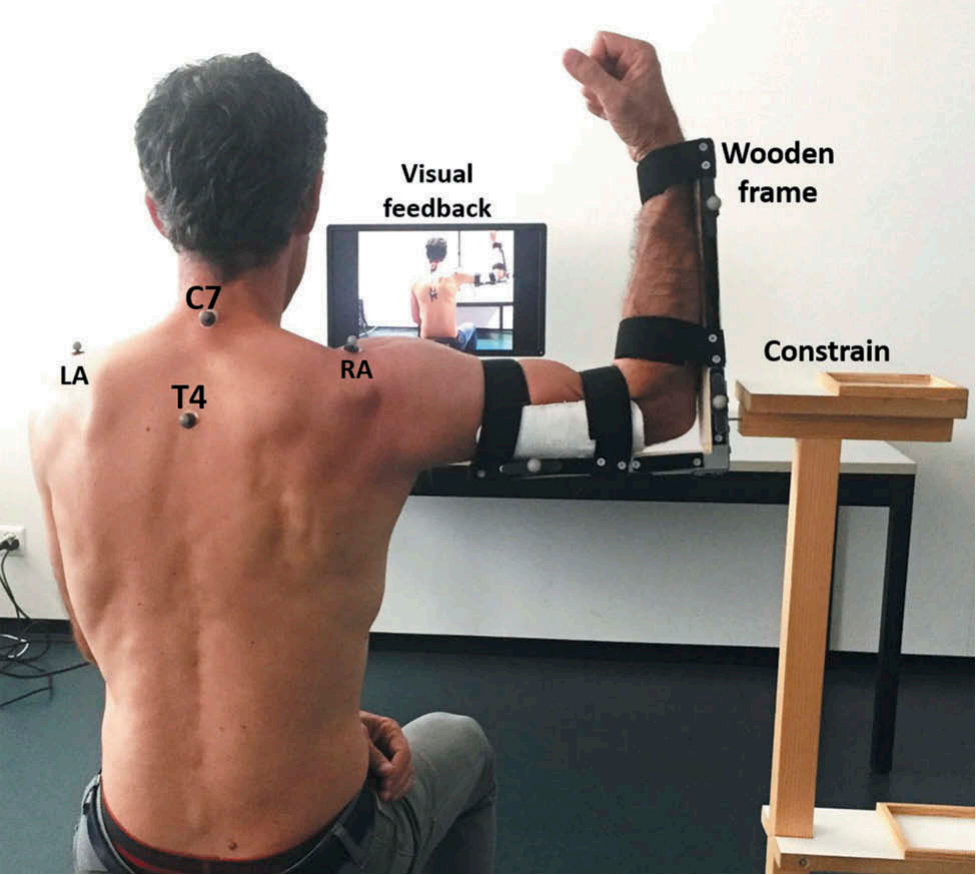
Position of the markers on a subject during the measurements. The wooden frame is fixed to the arm and upper arm with Velcro straps. Optical markers are taped to the wooden frame, and in correspondence of the anatomical landmarks. The constrain used for the third condition is also visible.

An ‘L’ shape wooden frame was built to fix the lower and the upper arm with an angle of 90 degrees. The wooden frame was composed of two sticks fixed with metal screws, one concave plastic support to keep the upper arm firmly attached to the frame. The wooden frame was fixed with four Velcro straps to the subjects arm and forearm to maintain 90 degree flexion of the elbow for the entire duration of the experimental session. The experimenter ensured that the position of the wooden frame and the tightness of the straps was appropriate. In case the arm could slide in the frame, the Velcro straps were repositioned and tightened to maximally reduce the possibility of movement. One reflective marker was positioned on each shoulders, positioned above the acromial edge to have a reference anatomical landmark for the position of the HA. Two markers were positioned on the sternum (upper and lower edge of the sternum), another on the skin corresponding with the spinous process of C7, one on T6 vertebra to create the thorax reference frame (ISB guidelines) that was later used as a reference frame for the computation of the HA of the humerus. Seven markers were positioned on the wooden frame fixed to the upper arm (see Figure 1), The redundancy of information of the markers on the reference frame was necessary to avoid loss of information due to shadow effects, since we had only six cameras available in our laboratory. The subject was seated relative to the room coordinate system so that the X-axis was transverse, the Y-axis was anterior–posterior and the Z-axis was vertical (Cole et al. 1993).

### Procedures

In sitting, the participants were asked to perform internal and external rotation of the shoulder to the end of the available range (see Figure 1). The arm was abducted to 90 degrees, the starting position for the movements was with the forearm vertical, so that the L shape wooden frame was lying on the frontal plane. The subject were then asked to perform 10 cycles moving from end range internal to end range external rotation, back to internal rotation, keeping the elbow approximately in the same position. In this way, the theoretical axis of rotation of the arm should be in the frontal plane and parallel to the ground, along the X-axis.

The subjects were asked to perform smooth cyclic movements to reach the maximum range of motion (RoM). The operator instructed each participant to maintain the upper arm in the abducted position and to rotate the arm until a feeling of discomfort was experienced in the shoulder at the end of each direction.

The 10 cycles of full rotations were performed to generate a larger dataset for HA computation, the first and the last cycles were removed from the analysis to reduce any starting and ending effects.

The 10 cycles of movements were performed in three different conditions:

1. Blindfolded. The subjects were asked to wear a black mask covering their eyes. The operator helped the participants to position the arm in the starting position (with the forearm vertical) and ensured that the elbow was maintained at 90 degrees abduction of the shoulder. If the shoulder angle changed during the data collection the operator would stop the data collection and ask the participant to start again. None of the subjects had to repeat the series more than two times.
2. With visual feedback. The participants were asked to keep their eyes open and focus on a computer screen where they could see the output of a camera positioned behind their back. In this way, the subjects could correct in real time any deviations of the arm, keeping the angle of 90 degrees of shoulder abduction as steady as possible.
3. With a mechanical constraint. The arm frame edge was locked to a spherical joint fixed to a wooden support. In this way, the subjects did not have to hold the weight of their arm, as it was supported.

In all three conditions, the participants were asked to sit tall on the edge of the chair and to keep the trunk as steady as possible.

Participants were asked to indicate hand dominance. The dominant hand was defined as the hand reported to be preferred for throwing a ball. The protocol was repeated for both arms in a randomized order.

### Signal processing

The cluster of four markers on the chest and back of the subjects was identified and analyzed as a rigid body using the SVD technique (Cappozzo et al. 1995). The trunk position was used as a reference frame, thus all the markers were transposed according to its instantaneous position to reduce the artifacts due to small oscillations of the subjects. The seven markers on the wooden frame were also identified as a rigid body and the arm rotations were analyzed with the HA technique (Söderkvist and Wedin 1993).

### HA computation

For each time frame the angle of the arm with respect to its initial position was computed. The angle was computed with the HA technique, thus as the angle of rotation about an axis to have the rigid body moving from the initial frame (F_0_) to the actual position at frame (F_i_).

Each of the instantaneous HA was computed using angle steps of 10 degrees. The choice was suggested by a preliminary study (Cescon et al. 2013) and was a compromise between smaller steps that could lead to higher variability, and bigger steps who would carry less information about the shoulder stability.

Once the group of HA was computed from the remaining cycles, a series of sagittal planes were defined for each of the two shoulders. Each plane was perpendicular to the X-axis and passing through a specific point as described in the following paragraphs.

### Mean distance of HA

For each of the defined planes, we computed the coordinates of the intersection points of the group of HA with the plane. The barycenter of the intersection points was then computed as the average value of the coordinates of the points. The distance between each point and the barycenter was then computed and the distribution of these distances was analyzed. These distances are assumed to have a Rayleigh distribution (continuous probability distribution for positive-valued random variables), thus the expected value (MD) could be obtained with the following equation:

$$MD=\frac{\sum_{i}^{N}d_{i}}{N}=\sigma \sqrt{\frac{\pi }{2}}=\sqrt{\sigma_{y}\sigma_{z}}\sqrt{\frac{\pi }{2}}$$

**Figure 2.**
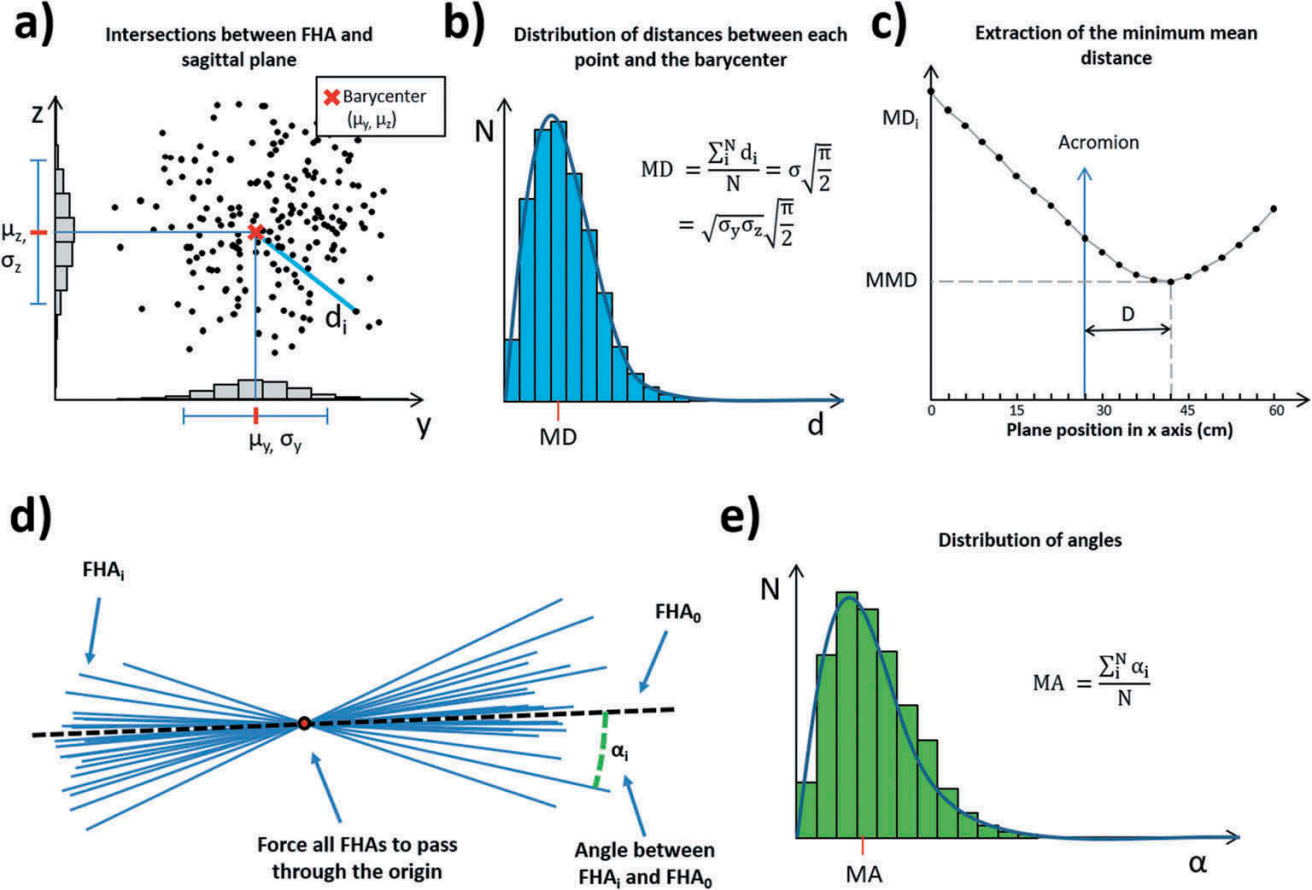
(a) Distribution of the intersection points of the HA with the sagittal plane. (b) Distribution of the distances between each point and the barycenter. The distribution of the distances is assumed to have a Rayleigh distribution. (c) Extraction of the minimum value (MMD) from the series of MD computed for each plane. (d) Representation of the HA translated in space to pass through the origin for the computation of the angles with respect to the HA_0_. (e) distribution of the angles between each HA and the HA0.

Where d_i_ is the distance of each point from the barycenter, and σ_y_ –σ_z_ are the standard deviations of the distribution of each of the two coordinates of the points in the plane ZY (see Figure 2(a,b)).

The procedure was performed for 21 planes spaced 3 cm in the X direction, from the sagittal plane (defined by the equation X = 0) to the plane defined by the equation X = 60 cm (X = −60 cm for the left arm). The choice of number of plane and spacing was a compromise due to the need of covering the entire length of the upper arm and constrain and to have a sufficient number of planes to identify the X coordinate of the minimum MD.

For each plane, *i* the MD*_i_* value was computed and the minimum value of MD was identified (MMD) as well as its coordinate along the X axis. The distance (D) between the identified planes and the marker on the acromion (see Figure 2(c)) was considered for further analysis. The technique was similar to the method described previously for identifying the minimum convex hull area (Cescon et al. 2014).

### Mean angle of HA

The procedure for the analysis of mean angle (MA) is described in the following paragraphs. The unit vector of each HA was extracted, and the mean unit vector was computed. The mean angle of the HAs with respect to the mean unit vector (see Figure 2(e)) was computed using the following equation:

$$MA=\frac{\sum_{i}^{N}\alpha_{i}}{N}$$

**Figure 3.**
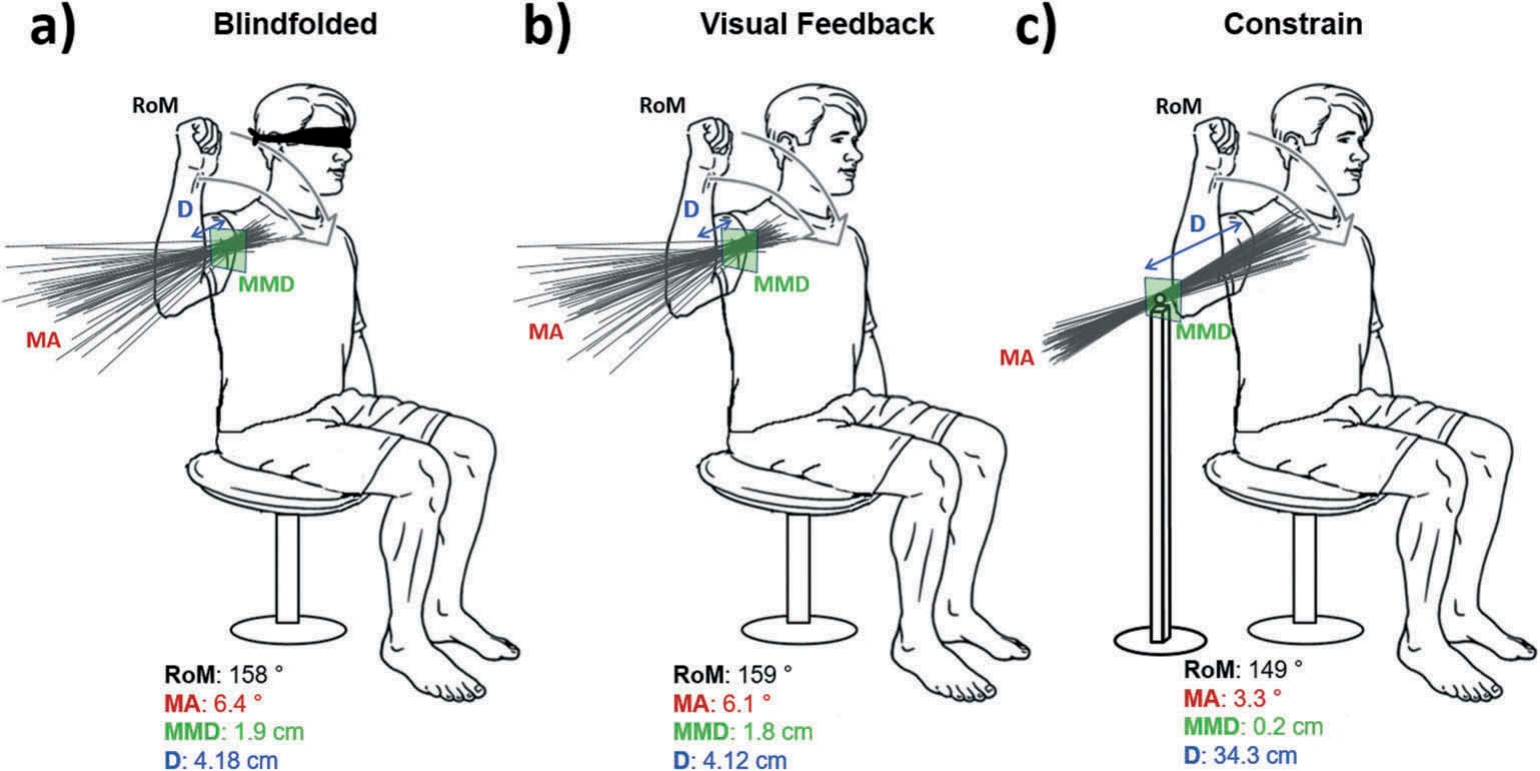
Subject position during the internal and external rotations of the shoulder. The HA are shown for the three conditions: (a) blindfolded, (b) visual feedback and (c) mechanical constrain. The plane corresponding to the MDD is shown in green. The four extracted parameters (RoM, MA, MDD and D) are shown for a representative subject during the three movements. As expected the HA are passing through the mechanical constrain when present.

Where α*_i_* is the angle between each of the HA unit vector and the mean unit vector. In addition, the RoM of each arm was measured in degrees for each of the three conditions (see Figure 3).

### Statistical analysis

The variables used for the statistics were the RoM, MA, MDD, and D, computed for each side (dominant and non-dominant) and each of the three movements (blindfolded, visual feedback, and constrained).

Comparison between dominant and non-dominant side was performed using Mann Whitney U-Test for each condition. Comparison between the variables during the three conditions was performed using the Kruskal–Wallis test.

Correlation between variables was performed using the Pearson’s correlation coefficient and the coefficient of determination R^2^. Significance level was set to α = 0.05.

## Results

Four HA parameters in three different conditions have been estimated for all volunteers and their distribution is summarized in Table 1.

**Table 1.** Summary of the four variables computed during the three movements on non-dominant and dominant side. Values are presented as mean ± standard deviation.

	Non-dominant side			Dominant side		
	Blindfolded	Visual Feedback	Constrain	Blindfolded	Visual Feedback	Constrain
RoM (°)	148.3 ± 18.0	147.4 ± 17.9	156.6 ± 17.3	143.0 ± 17.2	148.6 ± 20.4	157.2 ± 16.7
MA (°)	8.44 ± 1.40	7.82 ± 1.36	4.55 ± 0.64	6.55 ± 1.15	6.15 ± 1.12	3.43 ± 0.71
MDD (cm)	2.09 ± 0.51	2.03 ± 0.43	0.48 ± 0.07	1.69 ± 0.34	1.70 ± 0.38	0.25 ± 0.02
D (cm)	3.11 ± 1.97	4.18 ± 2.34	33.40 ± 4.31	2.34 ± 1.75	3.86 ± 3.46	35.27 ± 2.18

Only two participants were left hand dominant.

**Figure 4.**
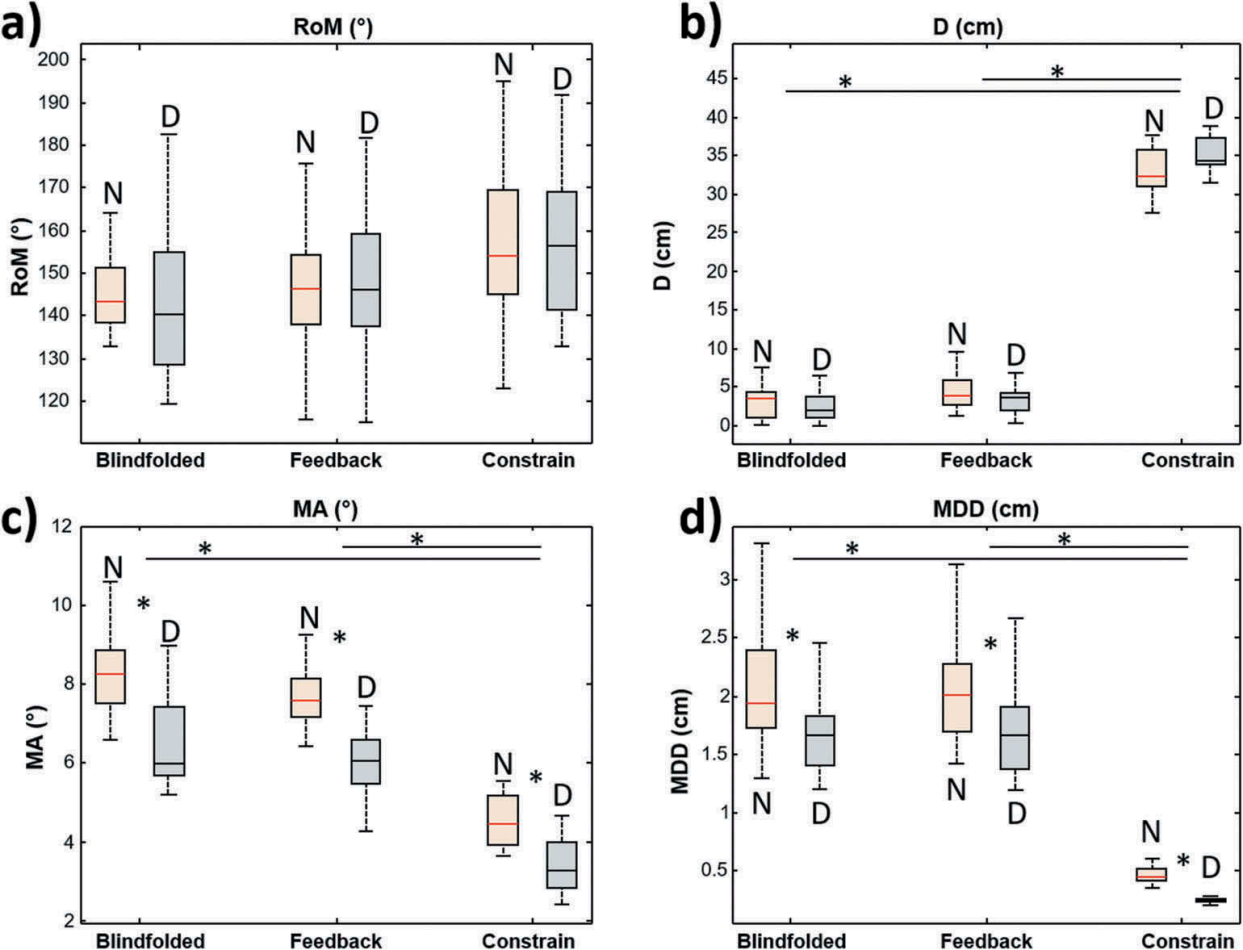
Comparison of the investigated parameters (RoM, MA, MMD and D; panels a, b, c and d respectively) in the three conditions (Blindfolded, feedback, and constrain). The data are shown for left and right side. Stars indicate statistical differences.

No significant differences were observed in the range of movement for each condition between dominant and non-dominant side. The mean angle of the axis distribution was significantly lower on the dominant side compared to the non-dominant in each of the three conditions. The axis dispersion (MDD) was also lower for the dominant side compared to the non-dominant side in each of the three conditions (see Figure 4).

None of the four parameters was significantly different between the first two conditions (blindfolded and visual feedback). A significantly lower MDD (P < 0.01) and significantly lower MA (P < 0.01) were observed in the condition with constrain (see Figure 4).

**Figure 5.**
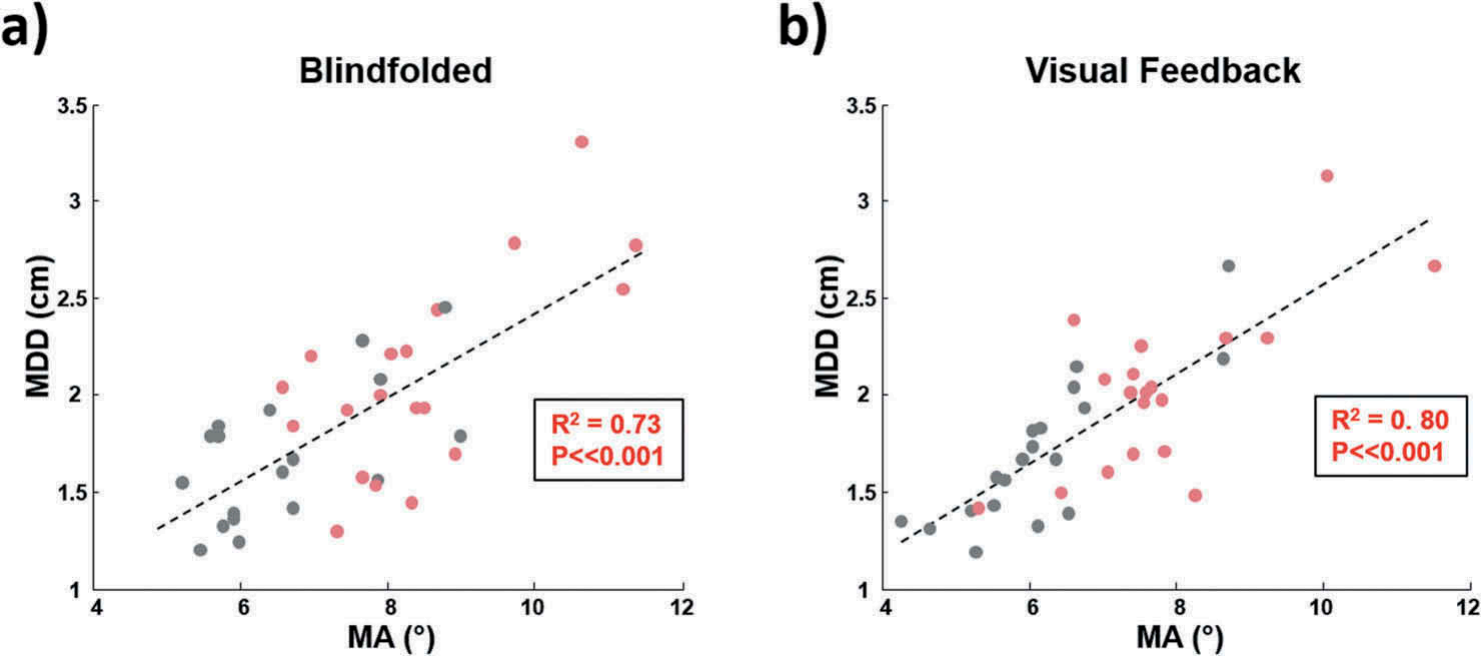
Scatter plots of MMD in the two conditions (Blindfolded and visual feedback; panels a and b respectively). Correlation between the variables is statistically significant in both conditions and the R2 values are indicated.

A significant correlation was observed between MA and MDD in the first two conditions (R^2^ = 0.53, p ≪ 0.001 for the visual feedback, R^2^ = 0.63, p ≪ 0.001 for the blindfolded condition; see Figure 5).

For both arms, the full range of shoulder motion was approximately 150 degrees. The distance D was between 3 and 4 cm during the first two tasks while was about 35 cm in the constrained task as expected, since the mechanical constrain (spherical joint) forced the arm to rotate around lines having the same intercept located in the center of the constrain. The mean angle was about 6–8 degrees during the first tasks while it was reduced to half its value during the constrained task. The MDD was about 2 cm during the first two tasks and less than 0.5 during the constrained task as expected.

No difference in range of motion during the three conditions was found. This was important as it allows for the comparison of the HA parameters of the three conditions.

The first two conditions (blindfolded and visual feedback) did not show significant differences in any of the parameters investigated. This suggests that in participants without symptoms visual input does not improve the ability to perform the requested task when proprioception is not impaired, as was the case in the present study. For people with symptoms, their proprioception may be altered (Urra et al. 2015; Bardal et al. 2016), and the HA parameters may show different values in these two conditions.

## Discussion

Previous studies have aimed to evaluate the axis of rotation of the shoulder (Stokdijk et al. 2000; Lempereur and Rémy-Néris 2014), in particular Stokdijk and collaborators described a method to analyze the intercept or the instantaneous helical axes computed during shoulder movement. The equipment used for the analysis was an electromagnetic system, which may be affected by distortions and measurement errors (Cescon et al. 2015). To our knowledge, this is the first study to quantify the kinematics of the shoulder using HA approach during a dynamic task, which requires an important contribution of both active and passive stabilizers of the shoulder. Those tasks have been selected by speculating in their capacity to highlight the kinematics’ differences between a stable and an unstable shoulder.

The significant difference between the two sides suggests that the dominant shoulder may be more ‘stable’, since the dispersion and the angle of the helical axis is lower. Smaller values of MDD and MA correspond to a rotation that is more similar to a pure hinge, where theoretically MDD and MA would be equal to zero. The observed difference could be explained because of better motor control due to the larger use (Assi et al. 2013).

Due to routine functional tasks, the dominant side may be better able to perform rotational movements, and this could lead to a smoother movement, that is observed in the lower values of MA and MDD for the dominant side compared to the non-dominant side.

Another potentially relevant finding was the lower MA during the third condition (constraint). This may be because in this condition the arm was constrained whereas during the first two conditions the elbow was not fixed. The MA during the constraint condition is not zero because the shoulder joint is not a perfect spherical joint, thus the finite helical axis angles change during the arm rotation. This suggests that the constrained condition may permit the assessment of glenohumeral joint stability as it fixes the distal position of the HA, but allows for the measurement of the variability in the proximal point of the HA. This hypothesis would require testing in future research.

The correlation between the MDD and MA is statistically significant in both conditions (visual feedback and blindfolded) with the percentage of explained variance being 53% and 63% for the two conditions respectively (Figure 5). This result suggests that these two parameters are complementary and are both useful to describe the impairments of the shoulder kinematics in terms of translation and orientation of the movement.

### Limitations

The results of the present study may be affected by some limitations both in technical aspects and in to the design of the study.

The first limitation is the type of movements chosen for the study. We focused only on the shoulder rotation with the arm abducted. The same analysis described in this article could be performed during other positions where the subject is asked to rotate the arm about any theoretical axis.

Another limitation is the wooden frame. Although it was built with light wood, its weight was not negligible with respect to the arm (200 g), and may have influenced the natural movement of the arm. We decided to use this frame to fix the elbow angle to 90 degrees, allowing the subjects to focus only on the shoulder joint during the movement.

The analysis demonstrated that the MDD during the constrained test was different from zero. Theoretically we expected to observe all the axis passing through the same point (the constrain center) because the arm was fixed, but probably the wooden support positioned on the ground was moving due to the weight and forces applied by the arm. The variability of the position of the axis is about 2–3 mm, thus also the value of MA extracted could be affected by the small movements of the support. This problem could be solved using a heavier and more stable support.

The results suggested a difference between the two sides, but we are unable to determine if this was due to side dominance because only two subjects were left dominant. We attempted to analyze the data according to left and right instead of side dominance, but of note the results did not change substantially. It would be relevant to analyze a larger group of left dominant subjects.

### Future perspectives

The next step after this study could be the evaluation of the proposed parameters in patients with shoulder instability, or the follow up of patients who have undergoing shoulder reconstruction and/or rehabilitation. The proposed technique could also be applied to other joints where is useful to evaluate stability, such as knees, ankles or elbows.

## Conclusion

Four parameters (RoM, MA, MMD, and D) that quantify the kinematics of the humeral rotations have been evaluated in healthy volunteers. The parameters appear to be sensitive and complementary in describing the humeral rotations during the three dynamic tasks of shoulder. The proposed approach is a promising method for the evaluation of the shoulder instability but further investigations with shoulder conditions are needed.
